# Treatment strategies for simple elbow dislocation** - **a systematic review

**DOI:** 10.1186/s12891-024-07260-0

**Published:** 2024-02-16

**Authors:** Franziska Lioba Breulmann, Sebastian Lappen, Yannick Ehmann, Martin Bischofreiter, Lucca Lacheta, Sebastian Siebenlist

**Affiliations:** 1 Department of Sports Orthopedics, Klinikum Rechts Der Isar, Technical University of Munich, Ismaningerstraße 22, Munich, 81675 Germany; 2 Department of Orthopedic Surgery, Ordensklinikum Barmherzige Schwestern Linz, Linz, 4010 Austria; 3 Department of Orthopedics and Traumatology, Klinik Diakonissen Schladming, Schladming, 8970 Austria

**Keywords:** Simple elbow dislocation, Simple elbow luxation, Treatment strategies, Elbow instability

## Abstract

**Background:**

Current treatment concepts for simple elbow dislocation involve conservative and surgical approaches. The aim of this systematic review was to identify the superiority of one treatment strategy over the other by a qualitative analysis in adult patients who suffered simple elbow luxation.

**Study design:**

A systematic review in accordance with the PRISMA guidelines and following the suggestions for reporting on qualitative summaries was performed. A literature search was conducted using PubMed and Scopus, including variations and combinations of the following keywords: elbow, radiohumeral, ulnohumeral, radioulnar, luxation, and therapy. Seventeen studies that performed a randomized controlled trial to compare treatment strategies as conservative or surgical procedures were included. Reviews are not selected for further qualitative analysis. The following outcome parameters were compared: range of motion (ROM), Mayo Elbow Performance Score (MEPS), Disabilities of the Arm, Shoulder and Hand outcome measure (Quick-DASH), recurrent instability, pain measured by visual analog scale (VAS) and time to return to work (RW).

**Results:**

Early mobilization after conservative treatment strategies showed improved ROM compared to immobilization for up to 3 weeks after surgery with less extension deficit in the early mobilization group (16° ± 13°. vs. 19.5° ± 3°, *p* < 0.05), as well as excellent clinical outcome scores. Surgical approaches showed similar results compared to conservative treatment, leading to improved ROM (115 vs. 118 ± 2.8) and MEPS: 95 ± 7 vs. 92 ± 4.

**Conclusion:**

Conservative treatment with early functional training of the elbow remains the first-line therapy for simple elbow dislocation. The surgical procedure provides similar outcomes compared to conservative treatment regarding MEPS and ROM for patients with slight initial instability in physical examination and radiographs. People with red flags for persistent instability, such as severe bilateral ligament injuries and moderate to severe instability during initial physical examination, should be considered for a primary surgical approach to prevent recurrent posterolateral and valgus instability. Postoperative early mobilization and early mobilization for conservatively treated patients is beneficial to improve patient outcome and ROM.

## Background

Elbow dislocations are common joint dislocations with an incidence of 6 per 100,000 [1, 2]. They can be classified as simple or complex types, with simple elbow dislocations showing no associated fractures and complex dislocations being characterized by additional bony injuries [3]. Elbow dislocations are particularly common in men as the result of an assault or sports and in women following a direct impact such as a fall from height [4–6].

The elbow is a highly congruent joint of high stability. The ulnohumeral joint as well as the medial and lateral collateral ligament complexes are considered primary static stabilizers. As part of the lateral collateral ligament complex, the lateral ulnar collateral ligament is restrained to varus and posterolateral rotatory instability [7]. Secondary stabilizers include the capsule, origins of the flexor common and extensor tendons and the radiocapitellar joint [8, 9]. Additional dynamic stabilizers, such as the anconeus, brachialis, biceps and triceps muscles, increase the stability of the elbow through compressive forces [10].

The main historically described mechanism causing elbow dislocation is a sequential disruption of the soft-tissue structures from lateral to medial, referred to as the Horii circle [11]. However, more recent studies describe the typical mechanism of injury to be a combined hyperextension with valgus force and axial loading [5].

The standard treatment for simple elbow dislocation has been described as conservative therapy with temporary immobilization of the joint after closed reduction and subsequent functional rehabilitation, while 2.3% of initially conservatively treated patients underwent surgery after a median time of 1 month [12]. Surgical treatment, on the other hand, is indicated for patients with high-grade soft tissue injuries and a tendency to redislocate to prevent chronic instability [13]. Schnetzke et al. showed that moderate joint instability after simple elbow dislocation leads to a significantly worse clinical outcome and more complications when initially treated conservatively. However, there is still no clear consensus on the standard procedure for simple elbow dislocation. Due to limited literature concerning treatment decisions for simple elbow dislocations, the aim of this systematic review is to compare common treatment strategies for simple elbow dislocation in adults followed by a qualitative analysis.

## Methods

### Literature search and inclusion criteria

A systematic review in accordance with the PRISMA guidelines and following the suggestions for reporting on qualitative summaries was performed [14–16]. A literature search was conducted using PubMed and Scopus, including variations and combinations of the following keywords: elbow, radiohumeral, ulnohumeral, radioulnar, dislocation, and therapy. All original articles investigating the treatment of elbow luxation were eligible for inclusion. Specific inclusion criteria were as follows: 1) original studies investigating acute simple elbow dislocations in adults, 2) randomized controlled trials, 3) original studies comparing conservative and surgical treatments, and 4) studies written in English, French or German. Studies that 1) were not available as full texts or 2) were retracted articles were excluded from further analysis. Conference abstracts were also not included. All records published before April 30th, 2023, were eligible for inclusion.

### Study selection, data extraction, and aggregation

Data were extracted by two reviewers (FLB, LL), and tables were created including information on first author, year of publication, study design, number of patients included, mean follow-up, type of intervention, control group, primary outcome parameters and secondary outcome parameters. In cases of imprecise, uncommon, unclear/conflicting, or missing descriptions of methods or participants, studies were excluded.

We defined the following scores as primary outcome parameters: Mayo Elbow Performance Score (MEPS) [17], Broberg and Morrey Score [18], the shortened version of the Disabilities of the Arm, Shoulder and Hand outcome measure (Quick-DASH) [19] and Oxford Elbow Score (OES) [20]. Secondary outcome parameters were defined as range of motion (ROM), recurrent joint instability, subjective pain measured by visual analog scale (VAS) and time to return to work (RTW).

### Grouping of studies and synthesis

To provide a structured qualitative summary, studies were grouped by two main categories: A) conservative treatment and B) surgical treatment. The validity of the reported findings was assessed using the categories of study design, number of patients included, median follow-up, type of intervention, control group, primary outcome parameters and secondary outcome parameters.

The certainty of the evidence was assessed using an evaluation of how directly the included studies addressed the planned applied methodology (measurement validity), the number of studies and the consistency of effects across studies. The risk of bias of the included studies was not assessed since only studies performing a randomized controlled trial or retrospective study were included. By strictly following the PRISMA guidelines to improve the quality of reporting, as well as reducing the risk of bias for selection by providing a structured approach. We qualitatively analyzed the included studies to evaluate which treatment method is most promising to achieve the primary and secondary outcomes.

### Statistical data analysis

We performed qualitative analysis of the included studies. Quantitative data is presented as mean ± SD. We presented *p* values given by the included studies. If a confidence interval is given in the included study, we reported confidence intervals of the studies.

## Results

### Literature search

**Fig. 1:**
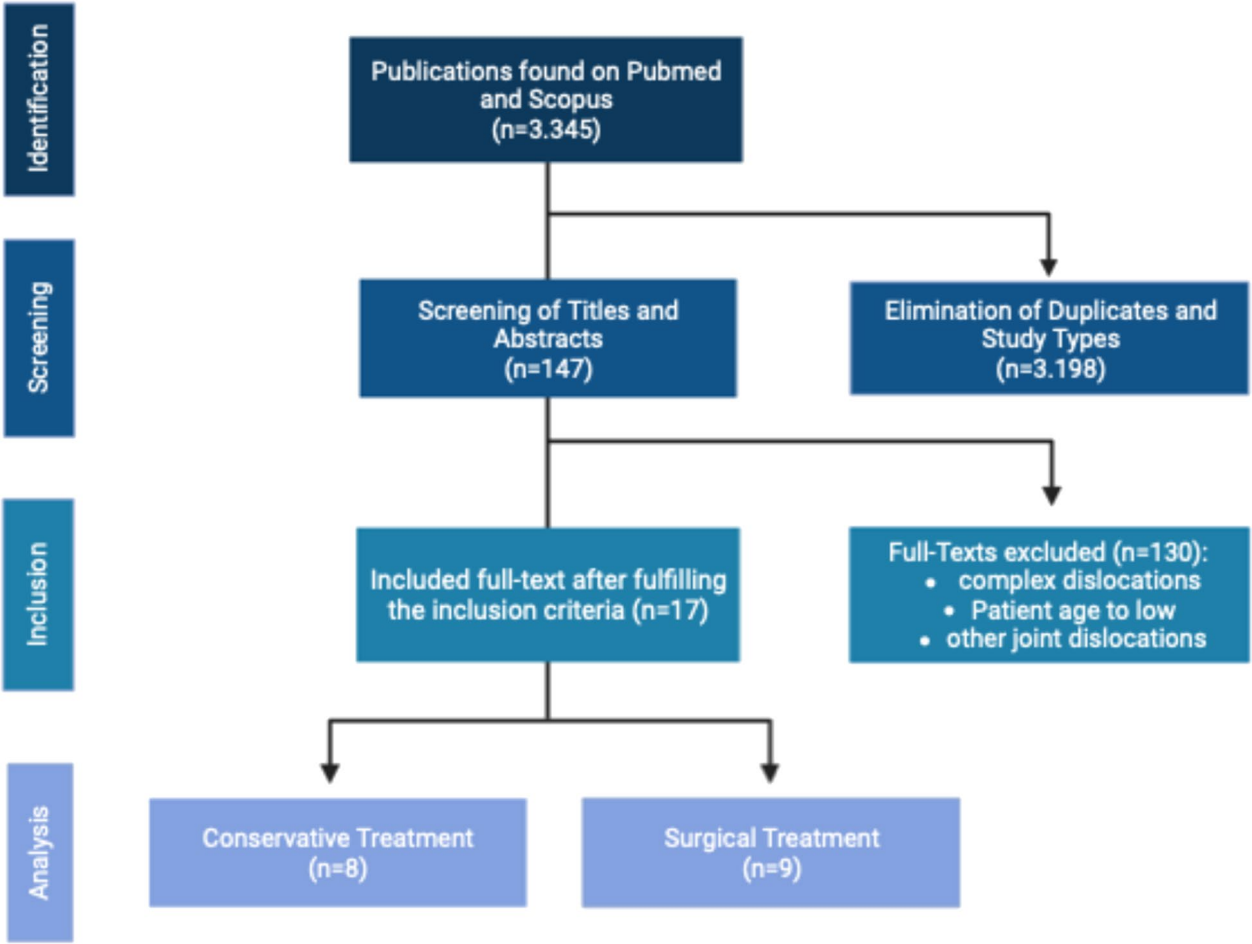
Flow chart of the literature search and included studies for qualitative and quantitative analysis. The systematic literature search identified 3,345 publications. Due to duplicates and the study type, 147 titles and abstracts were screened. By applying the inclusion criteria, 17 full-text articles were included for qualitative analysis

The search resulted in *n* = 3237 records on PubMed and *n* = 108 on Scopus (Fig. 1). Of 3345 records, 147 full text articles were screened: 55 full text articles found on PubMed and 92 on Scopus. Seventeen full-text articles were included after fulfilling the inclusion criteria.

The full text articles included in the qualitative analysis of simple elbow dislocations were divided into two groups: conservative treatment comparing early mobilization and immobilization on the one hand (Table 1) and surgical treatment strategies on the other hand (Table 2).

**Table 1:** Conservative treatment of simple elbow dislocations

Author (Year)	Studydesign (SD)	n	*Middle Follow-up (in months)*	Intervention (INT)	Control group (CG)	Primary outcome parameters	Secondary outcome parameters	Injuries following dislocation (ruptured ligaments)	Redislocations
De Haan, J. (2014) [21]	RCT	100	12	pressure bandage for 5–7 days and early functional treatment	plaster in 90-degree flexion, neutral position for pro-/supination for 3 weeks	No difference in Quick-DASH, MEPI, OES after 12 months; Quick-DASH after 6 weeks: improved by INT (*p* < 0.05)	Pain after 1 week: increased in CG (*p* < 0.05); ROM after 6 weeks: INT: CG = 121°: 102° (*p* < 0,05); Return to work: INT: CG = 8 d: 18 d (*p* < 0,05)	-	0
Iordens, G. (2017) [22]	RCT	100	12	early mobilization (*n* = 48): 4.0 (95% CI 0.9 to 7.1); at 6 weeks: arc of flexion and extension (121° (95% CI 115° to 127°)	3 weeks plaster immobilization (*n* = 52): 4.2 (95% CI 1.2 to 7.2); at 6 weeks: 102° (95% CI 96° to 108°)	*Quick*-DASH scores at 1 year: 4.0 (95% CI 0.9 to 7.1) points in INT group vs 4.2 (95% CI 1.2 to 7.2) in the CG group	Return to work: INT: CG: 10 vs. 18 d (*p* = 0,02)	INT:CG in type of dislocation: posterolateral 56/56%, posterior 17/19%, lateral 10/10%, posteromedial 6/6%, medial 0/2%	0
Von Lieshout, E. (2020) [23]	RCT	100	12	early mobilization (immediate motion exercises; *n* = 48)	3 weeks plaster immobilization (*n* = 52)	-	No significance in health-related quality of life in 1 year follow-up [EuroQoL-5D (EQ-5D: 0,88 (INT) vs. 0,89 (CG)) and Short Form-36 (SF-36 PCS (53 in INT and CG) and SF-36 MCS (55 and 56))], return to work INT: CG: 10: 18 d (*p* > 0.05)	-	-
Maripuri (2007) [24]	RCS	47	24–60	early mobilization	2 weeks of immobilization	Mean MEPI 83.8 (CG) vs. 96.5 (INT); quick-DASH 12.8 (CG) vs. 2.7 (INT) (*p* < 0.05)	return to work INT: CG: 6.6 weeks vs. 3.3 weeks	-	0
Mehlhoff (1988) [25]	RCS	52	34	early mobilization	Immobilization > 24 d	-	-	-	-
Schippinger (1999) [26]	RCS	45	62	early mobilization	immobilization > 3 weeks	Morrey Score: 56% excellent, 33% good result with less good results depending on the time of immobilization	Return to work: 36 patients	Patients with additional injuries following dislocation were excluded (e.g.: proc. Coronoideus fracture, lateral/medial avulsion)	-
Riel (1993) [27]	RCS	39	35–156	early mobilization	Immobilization 3–4 weeks	-	-	-	0
Eygendaal (2000) [28]	RCS	50	108	closed reduction of posterolateral dislocation (immobilization for 3 weeks)		residual valgus instability	degeneration in radiographs (ectopic ossification)	-	-

*SD* Study design, *RCT* randomized controlled trial, *RCS* retrospective comparative study, *PRS* prospective comparative study, *INT* intervention, *CG* control group, *DASH score* Disability of the Arm, Shoulder and Neck Score, *MEPI* Mayo Elbow Performance Index

**Table 2:** Surgical treatment of simple elbow dislocations

Author (Year)	Studydesign (SD)	n	*Middle Follow-up (in months)*	Intervention	Control group	Primary outcome parameters	Secondary outcome parameters	Redislocaction
Rafai, M. (1999) [27]	RCT	50	-	reduction followed by early mobilization (*n* = 24)	reduction followed by immobilization for 3 weeks (*n* = 26)	-	Extension deficit: INT: CG 4%: 19%	-
Wu, X. (2017) [29]	RCS	44	15	combined posterior lateral and anteromedial approach	Conservative treatment	MEPS: INT: CG postoperative 89.9: 50.4	Range of motion postoperative: flexion INT: CG 116.5°: 79.5°	-
Josefsson (1987) [30]	PRS	50	-	surgical treatment of the ligamentous injuries	nonsurgical treatment of the ligamentous injuries		extension deficit: INT: CG 18: 10; residual pain 50% in both groups	-
Duckworth (2008) [31]	RCS	14	27	lateral + medial ligamentous injuries	isolated surgery lateral	BMS: INT: CG 87.7: 87.2 (*p* < 0.05)	redislocation, residual subluxation	1
Kim (2013) [32]	RCS	15	9	surgical treatment of medial + lateral ligaments	isolated surgery lateral	MEPI: INT: CG: 82.9: 93.2 (*p* < 0.05)	Range of Motion: INT: CG 115°: 120° (*p* > 0.05)	
Micic (2009) [33]	RCS	20	32,5	surgical treatment of medial + lateral ligaments	isolated surgery lateral	MEPI: INT: CG: 91.9: 95 (*p* > 0.05)	ROM: INT: CG 106.8°: 121.1° (p > 0.05)	-
O Brien (2014) [34]	RCS	14	30	surgery of ligament ruptures in < 30 days after trauma	surgery of ligament ruptures in > 30 days after trauma	MEPI: INT: CG 100: 99.3 (*p* > 0.05)	ROM: INT: CG: 129.4°: 124.9° (*p* > 0.05)	-
Daluiski (2014) [35]	RCS	34	42	surgery of ligament ruptures in < 30 days after trauma	surgery of ligament ruptures in > 30 days after trauma	MEPI: INT: CG 90: 89 (*p* > 0.05)	ROM: INT: CG: 115°: 116°	0
Jeon (2008) [36]	RCS	13	27	Surgical treatment of medical + lateral ligaments	Isolated surgery later	MEPI: INT: CG 95.5: 86.7 (*p* > 0.05)	-	0

*SD* Study design, *RCT* randomized controlled trial, *RCS* retrospective comparative study, *PRS* prospective comparative study, *INT* intervention, *CG* control group, *DASH score* Disability of the Arm, Shoulder and Neck Score, *MEPI* Mayo Elbow Performance Index

### Conservative treatment

Eight studies analyzing conservative treatment strategies for simple elbow dislocations comparing early functional treatment against immobilization for 2 to 6 weeks were included [21–28].

Maripuri et al. observed improved range of motion (ROM) after conservative treatment with early mobilization compared to immobilization for up to 3 weeks after reduction, showing less extension in the early mobilization group: 16° ± 13° vs. 19.5° ± 3° (*p* < 0.05) (Table 3). In long-term follow-up, the early mobilized patients reported excellent clinical outcomes, with higher clinical outcome scores (MEPS) compared to the immobilized group: 96.5 (early mobilization) vs. 83.8 (immobilization) (*p* < 0.05) [24].

**Table 3:** Early mobilization vs. immobilization in operative treatment and conservative treatment

	**Parameters**	**Studies and amount of patients (n)**	**Statistical test:** **Mean + STD**
Operative Treatment: Early mobilization vs. immobilization	MEPI ROM	6 (140)	95 ± 7 vs. 92 ± 4 115 vs. 118 ± 2.8
Conservative Treatment			
Early mobilization vs. immobilization	Extension deficit	4 (347)	16 ± 13 vs. 19.5 ± 3

*MEPI* Mayo Elbow Performance Index, *ROM* Range of Motion, *RTW* Return to Work – Rate, *STD* Standard-Deviation

Iordens et al. and Maripuri et al. both showed superior Quick DASH scores in the early mobilization group after 12 and 24 months of follow-up [22, 24]. Iordens et al. observed a significant difference in RTW in the early mobilization group at 10 days compared to 18 days in the immobilization group (*p* < 0.05). Contrary to the results of Iordens et al., Maripuri et al. showed an RW rate of 6.6 weeks in the intervention group vs. 3.3 weeks in the control group (*p* < 0.05). Both studies did not observe any redislocation or recurrent instability in their early mobilization group.

Schippinger et al. compared functional outcome and ROM in patients with early mobilization to patients with immobilization of the elbow for at least three weeks [26]. At follow-up after 62 months, 56% of all patients showed excellent and 33% good results in the Morrey score, with inferior results shown in patients with longer immobilization times. Patients who have been immobilized for more than three weeks most likely showed a loss of terminal extension up to 30 degrees, and 36% showed a flexion deficit of 10 degrees. Recurrent instability was not reported in this study.

### Surgical treatment

Nine studies performed randomized controlled trials in patients who underwent surgical treatment [29–37]. Three of those nine studies compared surgical to conservative treatment [29, 30, 37].

The comparison of surgical treatment to conservative treatment showed worse MEPS for the conservative treatment group compared to the group that underwent surgery: 50.4 vs. 89.9 (*p* < 0.05). All patients showed complete rupture of the medial ligaments, and half of the patients showed additional rupture of the lateral ligament. They were randomly divided into surgically or conservatively treated groups. Josefsson et al. noticed a greater extension deficit in the surgically treated group compared to a conservatively treated control group (18° vs. 10° (*p* < 0.05)), while Rafai et al. showed less extension deficit in the intervention group: 4° vs. 19° (*p* < 0.05) [30, 37].

In surgical procedures, early mobilization after surgery favored ROM (115 vs. 118 ± 2.8) (*p* > 0.05) and led to improved clinical outcomes (MEPS): 95 ± 7 vs. 92 ± 4 (*p* < 0.05) [37]. Rafai et al. compared 24 patients who received closed reduction followed by early mobilization on the first day after reduction to patients who were treated with immobilization for 3 weeks after reduction (*n* = 26) [37]. As a result, they did not show a difference in extension deficits in the immobilization group compared to the early mobilization group (19 vs. 4%; *p* > 0.05). Additionally, they did not report any differences in pain, instability or recurrent dislocation of the elbow between the two groups.

Four studies compared surgical treatment in patients with either lateral plus medial ligament rupture or isolated lateral ligament injury. Two of those studies showed controversial MEPS after bilateral ligament injury repair compared to isolated lateral ligament repair with conservative treatment of MCL rupture: 82.9 vs. 93.2 (*p* < 0.05) [32] and 91.9 vs. 95 (*p* > 0.05) [33]. Jeon et al. reported no significant difference in MEPS in patients with bilateral ligament repair: 95.5 vs. 86.7 (*p* > 0.05) [36]. All studies reported a nonsignificant difference in ROM after bilateral ligament repair. The group with isolated lateral repair showed no difference in ROM with 120° and 121° (*p* > 0.05) compared to bilateral ligament repair of 115° and 106.8° (*p* > 0.05) [32, 33]. Jeon et al. recommend primary ligament repair for elbow dislocations with severe ligamentous injury, as it prevents late ligament repair and recurrent instability after conservative treatment. These results were supported by Micic et al., who showed improved clinical outcomes and the prevention of recurrent instability two to four years postoperatively [33].

Two studies analyzed the impact of time between injury and surgical treatment. O’Brien et al. and Daluiski et al. showed no difference in MEPS scores in the intervention groups (INT) that underwent surgery within 30 days after injury compared with the control group that underwent surgery more than 30 days after injury (CG): 100 (INT) vs. 99.3 (CG) (*p* > 0.05) [34] and 90 (INT) vs. 89 (CG) (*p* > 0.05) [35]. No difference in postoperative ROM was reported in INT compared to CG: 129.4° (INT) vs. 124.9° (CG) (*p* > 0.05) [34].

## Discussion

The main findings of this literature review are the following: 1. Early mobilization of the elbow should be performed in conservative treatment, as it leads to superior clinical outcomes with increased ROM and increased MEPS. Early mobilization showed equally low risks for complications as short-term immobilization. 2. Surgical therapy provides similar outcomes compared to conversative treatment as first-line therapy in bilateral ligament injuries. 3. Early mobilization after surgery favors ROM and MEPS and leads to superior RTW rates. 4. For patients with bilateral ligament injuries and severe instability, initial surgical treatment can prevent later instability of the elbow.

Our results align with a prior published review that emphasizes the significance of early functional treatment for the elbow following trauma or surgery [38]. However, our review further extends the scope by encompassing additional studies and providing a comprehensive analysis of research specifically focusing on surgical treatment for simple elbow dislocations.

### Conservative treatment

Conservative therapy is often considered the standard of care for simple elbow dislocations without severe ligamentous and bony injuries, as well as no instability in initially physical examination. However, there is still no clear consensus on the treatment details, such as the time of immobilization or functional treatment. Iordines et al. found no significant differences in long-term outcomes after early and late mobilization [22]. Maripuri et al., on the other hand, reported significantly superior long-term clinical scores after early mobilization but found no difference regarding health-related quality of life [24]. Other studies have shown a clear correlation between a longer duration of immobilization and a decrease in ROM [25, 39]. During preparation of the manuscript, we became aware of another long-term study by Mackinnon et al. [40]. This study shows excellent clinical long-term outcomes of simple elbow dislocation being treated by a short period of immobilization followed by early functional treatment [40].

Furthermore, no increase in complications could be seen due to early functional training. Most likely, the risk for loss of extension or flexion contracture increases with the time of immobilization [26]. The risk for redislocation has been described to be slightly lower in those undergoing early mobilization (1.3%) than in those undergoing short-term immobilization (2%) [41]. Overall, none of the included studies focusing on conservative treatment strategies, reported a redislocation. In addition, a lower prevalence of heterotopic ossifications was seen in patients after early mobilization [42]. In summary, due to the superior short-term and possibly long-term outcomes, for conservative management, early functional treatment should be performed according to the patient's pain tolerance to avoid complications such as elbow stiffness.

### Conservative vs. surgical treatment

Despite its name, simple elbow dislocations are complex injuries. This complexity raises the question of when surgery is indicated. The available data show that surgical repair of the ligaments as primary treatment leads to outcomes similar to those of conservative treatment [30].

Up to 10% of conservatively treated simple elbow dislocations develop complications requiring surgical treatment, with soft tissue stabilization and contracture releases being the most commonly performed revision surgeries [12]. However, efforts should be made to filter out those patients who benefit from primary surgical treatment to avoid often complex and burdensome secondary surgeries.

A study by Josefsson et al. showed that conservative treatment is associated with slightly less chronic instability [30]. In a follow-up of patients at an average of 24 years, they showed better long-term functionality with conservative treatment than with surgical procedures, without any recurrent dislocation or instability. However, this difference was not statistically significant. However, they reported a decrease in ROM in the conservatively treated group. As the study of Josefsson was performed in 1987, surgical procedures have improved in the last couple years with minimally invasive approaches and improved implants for ligament repair. This may lead to improved clinical outcomes and less recurrent instability after primary surgical procedures. However, an evaluation by Eygendaal et al. reported residual valgus instability in up to 48% of conservatively treated patients [28]. Schnetzke et al. emphasized that patients with initially moderate elbow instability show a significantly higher risk of developing complications and needing revision surgery [13]. The authors pointed out the importance of recognizing the early clinical and radiological signs of instability and including them in the decision-making process between conservative and surgical therapy. Red flags of persistent instability are instability under varus and valgus stress during clinical examination, drop sign defined as a humero-ulnar distance of at least 4 mm measured in lateral X-ray, and joint incongruence, determined in MRIs by an incongruence of the humero-ulnar and humero-radial joint lines [43–45]. Emphasis has been placed on taking into account injuries not only to the primary stabilizers but also to the secondary stabilizers, particularly those of flexor and extensor origins [3, 30, 46]. In particular, patients with severe soft-tissue damage requiring multiple reduction attempts are at risk for reductant instability and for requiring contracture release over time and might therefore benefit from early surgical treatment [12]. If surgery is performed, early mobilization is recommended immediately after surgery, as it favors ROM and MEPS and leads to lower RTW rates [22].

Conflicting results are reported regarding ROM. Josefsson et al. reported greater short-term extension deficits in surgically treated patients [30]. However, there was no significant difference over the long term. In contrast, Wu et al. reported superior elbow flexion and clinical outcomes after surgical treatment [29]. It has further been observed that patients report more pain in the short term after conservative treatment [47]. This could contribute to the development of reduced ROM in conservatively treated patients, as these patients might not be adequately exercising their elbow due to pain. Nevertheless, the amount of pain increases with prolonged immobilization of the elbow, and the ROM decreases. Therefore, patients might benefit from early mobilization to improve ROM and reduce pain. However, the data on this topic are still too limited for a reliable recommendation. Therefore, treatment decisions must be made on a case-by-case basis, taking into account the patient’s individual needs and risks.

### Surgical treatment

There is no clear consensus on when surgical treatment is indicated and on the postoperative procedure regarding immobilization and early functional treatment. The role of surgical stabilization of the elbow following simple elbow dislocation is controversially discussed in the current literature. Avoiding recurrent instability is the most important factor considered when deciding on conservative vs. surgical treatment strategies. As primary stabilators of the elbow, the collateral ligament complexes are mainly addressed in the evaluated studies. The degree of instability is greatly dependent on the extent of soft tissue damage, as well as on the loss of secondary dynamic stabilizers as the dynamic structures of the elbow increase the stability [10]. As Hackl et al. already concluded, there are limited data on the influence of severe soft tissue injury on the clinical outcome after elbow dislocation [48]. However, Schnetzke et al. showed worse clinical results in a group of patients who suffered moderate instability with angulation ≥ 10° compared to patients with only slight instability with joint angulation < 10° (MEPS 95.8 ± 9.0 vs. 90.0 ± 15.2 points; *p* = 0.154). The severity of ligamentous ruptures should be considered for treatment decisions.

Kim et al. reported on eight patients with simultaneous injuries to both the MCL and LCL after elbow dislocation and acute PLRI, which were treated with isolated LCL repair [32]. Residual medial instability was found in 2 of these patients, who, however, did not require further surgical treatment. The authors therefore argue that in these cases, LCL repair without MCL repair may be sufficient to prevent chronic PLRI and valgus instability. Furthermore, their study reported that patients with isolated lateral injuries showed better clinical outcomes but no difference in postoperative ROM compared to patients with combined LCL and MCL injuries. Micic et al., on the other hand, performed additional MCL repair in combined injuries and reported no statistically significant difference in clinical outcome or ROM in patients with isolated LCL injury or combined MCL and LCL injuries after surgical repair [33]. Due to the small number of cases in these studies, however, it is not possible to provide a definitive treatment recommendation. A randomized controlled trial between isolated lateral and combined medial and lateral repair would therefore be necessary to definitively answer this question.

O´Brien et al. and Daluiski et al. compared postoperative outcomes by the time of surgery. No significant difference in clinical outcome or ROM was found for early (within 30 days after trauma) or delayed repair (later than 30 days after trauma) [34, 35]. This reinforces conservative treatment of simple elbow dislocation as the primary first-line therapy since surgical treatment has the same chance of success if it is performed delayed after unsuccessful conservative therapy.

Additionally, one of the included studies, reported a single redislocation after surgery (1 out of 14) [31]. All other included studies did either not report any redislocations or did not see any recurrent instability in their patients. This should be assessed more in future studies and defined as a secondary outcome parameter for qualitative analysis of this treatment strategy.

### Limitations

There are several limitations in this literature review. First, the group sizes differ between the studies focusing on conservative treatment and those focusing on surgical treatment. This bias was reduced by performing qualitative analysis on those studies and comparing these results. Second, the studies focusing on surgical treatment differed in their chosen comparison: bilateral ligament repair or isolated lateral repair, immobilization after surgery, ligament repair or time of surgery. Therefore, these different studies using the same intervention and control were analyzed together, without comparing all studies that performed surgery together. Third, few randomized controlled trials are available to analyze treatment strategies for simple elbow dislocation, and a homogenous patient profile is rare. This review aims to provide an overview of the recent literature and provide recommendations for the treatment of simple elbow dislocations. Overall, the within this systematic review included studies show a high heterogeneity within the reported surgical techniques and follow-up times. The diversity in surgical techniques and follow-up durations among the studies represent a challenge in analyzing and comparing the findings. In the future, further investigations into treatment strategies are needed to implement evidence-based guidelines for simple elbow dislocation treatment and classification systems for simple elbow dislocations.

## Conclusion

Conservative treatment with early functional training of the elbow remains the first-line therapy for simple elbow dislocation. The surgical procedure provides similar outcomes compared to conservative treatment regarding MEPS and ROM for patients with slight initial instability in physical examination and radiographs. People with red flags for persistent instability, such as severe bilateral ligament injuries and moderate to severe instability during initial physical examination, should be considered for a primary surgical approach to prevent recurrent posterolateral and valgus instability. Postoperative early mobilization and early mobilization for conservatively treated patients is beneficial to improve patient outcome and ROM.

## Data Availability

All data generated or analyzed during this study are included in this published article.

## Abbreviations

ROM: Range of motion
MEPS: Mayo Elbow Performance Score
Quick-DASH: Disabilities of the Arm, Shoulder and Hand outcome measure
VAS: Visual analog scale
RW: Return to work
OES: Oxford Elbow Score
INT: Intervention Group
CG: Control Group
MCL: Medial collateral ligament
LCL: Lateral collateral ligament
SD: Study design
RCT: Randomized controlled trial
RCS: Retrospective comparative study
PRS: Prospective comparative study
MEPI: Mayo Elbow Performance Index
